# Key Considerations for the Treatment of Advanced Breast Cancer in Older Adults: An Expert Consensus of the Canadian Treatment Landscape

**DOI:** 10.3390/curroncol31010010

**Published:** 2023-12-26

**Authors:** Emily B. Jackson, Lauren Curry, Caroline Mariano, Tina Hsu, Sarah Cook, Rossanna C. Pezo, Marie-France Savard, Danielle N. Desautels, Dominique Leblanc, Karen A. Gelmon

**Affiliations:** 1 BC Cancer Vancouver Centre, 600 West 10th Avenue, Vancouver, BC V5Z 4E6, Canada; lauren.curry@bccancer.bc.ca (L.C.);; 2 Department of Medicine, University of British Columbia, Vancouver, BC V6T 1Z3, Canada; 3 The Ottawa Hospital Cancer Centre, Ottawa, ON K1H 8L6, Canadamsavard@toh.ca (M.-F.S.); 4 Department of Medicine, University of Ottawa, Ottawa, ON K1H 8L6, Canada; 5 Tom Baker Cancer Centre, Calgary, AB T2N 4N2, Canada; 6 Department of Medicine, University of Calgary, Calgary, AB T2N 1N4, Canada; 7 Sunnybrook Odette Cancer Centre, Toronto, ON M4N 3M5, Canada; rossanna.pezo@sunnybrook.ca; 8 Department of Medicine, Division of Medical Oncology, University of Toronto, Toronto, ON M5S 1A1, Canada; 9 Department of Internal Medicine, Rady Faculty of Health Sciences, University of Manitoba, Winnipeg, MB R3E 3P4, Canada; ddesautels@cancercare.mb.ca; 10 Paul Albrechtsen Research Institute, CancerCare Manitoba, Winnipeg, MB R3E 0V9, Canada; 11 Centre Hospitalier Universitaire de Québec, Université Laval, Québec, QC G1V 0A6, Canada

**Keywords:** breast cancer, older adults, systemic therapy, toxicity, expert consensus

## Abstract

The prevalence of breast cancer amongst older adults in Canada is increasing. This patient population faces unique challenges in the management of breast cancer, as older adults often have distinct biological, psychosocial, and treatment-related considerations. This paper presents an expert consensus of the Canadian treatment landscape, focusing on key considerations for optimizing selection of systemic therapy for advanced breast cancer in older adults. This paper aims to provide evidence-based recommendations and practical guidance for healthcare professionals involved in the care of older adults with breast cancer. By recognizing and addressing the specific needs of older adults, healthcare providers can optimize treatment outcomes and improve the overall quality of care for this population.

## 1. Case

Ms. L is a 91-year-old female retired hairdresser, living in an urban setting in Canada without any extended health benefits. Widowed, she now lives alone in a condominium she has owned for 30 years. Her two children live within a few hours’ drive. Ms. L typically ambulates independently. She socializes with nearby friends or family a few times per week.

Ms. L has recently been diagnosed with advanced breast cancer, having been presented to the hospital after a fall with imaging revealing widespread bony metastatic disease. Further workup confirmed a primary breast lesion and locoregional lymphadenopathy. There was no evidence of visceral metastases. Standard laboratory workup was all within normal range. She is otherwise healthy, with no other major comorbidities.

The biopsy of the primary breast mass reports invasive carcinoma of no special type. In the following sections, we review core considerations for Ms. L’s care, including discussion of systemic management options for all possible breast biomarker outcomes.

## 2. Background

The care of older adults with breast cancer is challenging and healthcare providers are not always well-equipped to manage the unique needs and concerns of these patients. With both the aging of the population and more effective and better tolerated treatments, older adults comprise a significant proportion of persons seen in oncology clinics. Breast cancer incidence is expected to almost double in Canada between 2012 and 2042, with the most significant increase projected amongst the older adult population. One recent model projected an increase in breast cancer incidence amongst individuals aged 75–84 from 3293 cases in 2013 to 8194 cases in 2042 (2.5× increase). In adults ages 85 or more, the projected incidence increased from 1697 cases in 2013 to 5004 cases in 2042 (2.9×) [1]. Older adults with cancer are more likely to have comorbidities and experience functional decline [2,3]. They are also likely to have more varied priorities for their care, with some valuing longevity while others value quality of life and/or less treatment burden. Therefore, to promote optimal treatment for older adults with breast cancer, clinicians must dedicate special attention to these crucial factors affecting these patients’ lives, combining it with knowledge regarding disease biology and treatment options.

## 3. Oncologic Assessment in Older Adults

The recent American Society of Clinical Oncology (ASCO) guideline for Geriatric Oncology recommends that all patients ≥ 65 years old who are receiving chemotherapy should have a brief geriatric assessment (GA) performed to identify vulnerabilities not routinely assessed in a standard oncology clinic setting [2]. GA and management of identified deficits has been shown to decrease moderate to severe chemotherapy toxicity and improve completion rates of chemotherapy. It may also result in improved quality of life in those receiving systemic therapy [4,5,6,7]. Studies have shown that GA can be completed through self-administered surveys and/or electronically with minimal involvement from clinic staff other than for cognitive and objective physical functioning testing [8,9]. The ASCO guideline recommends that, at minimum, a brief GA should include an assessment of functional capacity by enquiring about instrumental activities of daily living (iADLs), comorbid illnesses and general health, falls risk, depression (using the Geriatric Depression Scale), cognition (screen using the Mini-Cog or Blessed-Orientation-Memory-Concentration test), nutrition and unintentional weight loss [2]. ASCO has also recently released an updated guideline outlining a pared down “Practical Geriatric Assessment” to facilitate utilization of GA in the clinical setting [10].

There are validated screening instruments that can be easily incorporated into an oncology clinic setting to help clinicians evaluate patients who may benefit from a comprehensive geriatric assessment (CGA) [11]. The two most widely utilized screening GA tools are the Geriatric-8 (G8) screening tool and the Vulnerable Elders Survey-13 (VES-13), although several others exist [12].

Ultimately GA attempts to more comprehensively and better characterize older adults with cancer, enabling clinicians to help provide better recommendations and improve outcomes. In this review, we offer an approach to treatment decision making in older adults with breast cancer.

## 4. Key Treatment Considerations in Older Adults

**Figure 1:**
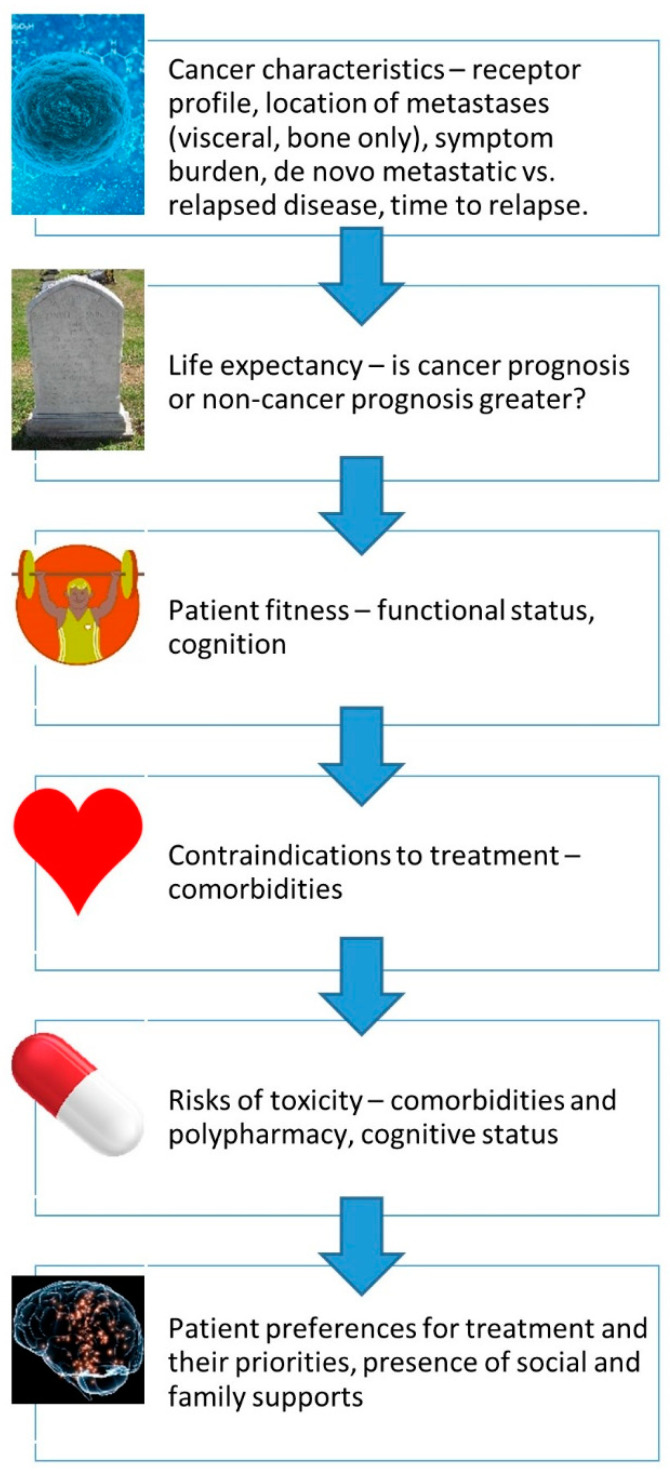
Approach to core considerations when assessing an older adult with cancer.

Older adults become more diverse with increasing age. While some older adults are very fit, others are more vulnerable or frail. Older adults are at risk of both overtreatment and undertreatment. Decisions regarding treatment options and recommendations must be based on a patient’s assessed fitness, vulnerabilities, frailty, life expectancy and personal goals, and not based on chronological age. In general, fit older adult patients should be offered standard of care therapy regardless of age [13]. Those with symptomatic breast or axillary masses should be considered for locoregional management (palliative intent radiotherapy or surgery) after multidisciplinary consideration as appropriate, regardless of chronological age. Less fit individuals or those with relevant comorbid illnesses may require modified treatment. This approach must then be combined with a shared decision-making process that respects a patient’s values and goals for their health care (Figure 1).

Older men diagnosed with breast cancer seem to have similar survival to older women, and improved survival compared with younger men [14]. There are no unique evidence-based treatment recommendations for older men, and guidelines suggest treating older patients the same regardless of biological sex.

### 4.1. Functional Status, Comorbidities, and Life Expectancy

Balancing efficacy against toxicity is a particular challenge in older adults who have diminished physiological reserves and increased comorbidity burdens. This problem is compounded by the underrepresentation of older adults in clinical trials, resulting in less evidence informing optimal drug selection, dosing, and monitoring for this unique and evolving patient population [15,16,17].

Assessing a patient’s current abilities and limitations is important to better predict the adverse effects of treatment, including its effect on functional status. Decreased functional reserve is a core component of frailty, and leads to increased vulnerability to adverse outcomes from treatments and the cancer disease process itself. Patients with poor function at baseline are at higher risk of functional decline with treatments such as chemotherapy [18]. Function can be assessed through subjective measures on history as well as through objective measures. Subjective measures of function include a patient’s ability to perform basic activities of daily living (i.e., transferring, ambulating, toileting, bathing, dressing) and instrumental activities of daily living (i.e., cooking, groceries, cleaning, laundry, medication management). A history of falls, particularly within the last 6 months, can predict future adverse events, both oncologic (i.e., risk of moderate to severe chemotherapy toxicity) and non-oncologic (i.e., hip fractures). Objective markers of function include tests of gait speed, strength, and stamina. Examples of these include the 4-metre walk test, Timed Up and Go test (TUG), the Short Physical Performance Battery, and grip strength. Patients with slower objective markers of gait speed are at risk of earlier mortality and functional decline [18,19,20]. Additionally, potential communication challenges including hearing impairment and language barriers should be identified and addressed during this process.

Comorbidities are important both to assess for tolerance and contraindications to treatment, as well as to understand competing risks for mortality, both of which may warrant consideration for modified and/or less intensive oncologic treatments. Medical comorbidities in cancer patients are associated with poorer survival, increased treatment toxicity and treatment discontinuation, and increased risk of hospitalizations [21,22,23,24,25]. Obesity is an important comorbidity and risk factor for increased rate of complications with cancer treatments, which must be considered. While data suggest that some systemic therapies may be less effective in obese individuals, there are no specific treatment recommendations for this patient population. Furthermore, while dose capping is generally not recommended, practitioners should exercise great caution and consider individualized dose modification in obese older adults. Using a validated tool, such as the Cumulative Illness Rating Scale-Geriatrics, allows for objective quantification of the severity or significance of a given comorbidity [26,27,28].

Life expectancy also plays a crucial role in treatment decision-making. Recognizing that older adults may have competing mortality risks, it is essential to assess life expectancy when considering the optimal treatment approach. Life expectancy can be estimated using validated tools such as the Lee Index and Schonberg Index, which can be found at eprognosis.org [29]. The time frame of relevance (e.g., 1 year, 5 years, 10 years) depends on various factors including the biology of the cancer, its stage, and when recurrence and/or cancer related complications and symptoms may emerge. For example, a hormone sensitive, human epidermal growth factor receptor 2 (HER2) negative 2 cm cancer may not be life limiting or cause local symptoms in a patient with a limited life expectancy while a 5 cm triple negative breast cancer could reasonably be expected to cause symptoms or limit survival over the near term. Individuals with a short life expectancy may not derive benefit from standard treatment options, such as surgical resection, and modified management including observation, radiation or endocrine therapy may be more appropriate. On the other hand, older adults with a longer life expectancy may derive significant benefits from intensive treatment and should receive standard of care treatment if they can tolerate it and the treatment aligns with their goals. Thus, individualized treatment plans accounting for a patient’s life expectancy and overall health status are vital for optimizing outcomes in older adults with breast cancer.

These domains change with time due to the underlying cancer, treatments received or other significant health events (i.e., a fall, stroke, myocardial infarction) and should be re-assessed at treatment decision points and/or after significant changes in health. Longitudinal reassessment will ensure future lines of therapy are appropriate and provide meaningful benefit.

### 4.2. Cognitive Assessment

The number of people in Canada living with cognitive impairment is rising, mainly due to growth of the population aged 65 and older [30]. The Public Health Agency of Canada estimates that 7.1% of people aged 65 of older were living with dementia in 2013–2014, and the prevalence reached 25% for those 85 and older.

Cognitive impairment is often under-diagnosed in the cancer population but is important to cancer management. Studies reporting the prevalence of cognitive impairment in patients with cancer vary greatly in their results, but most range between approximately 40–60% [31,32,33,34]. Cognitive impairment may be associated with an increased risk of toxicity from cancer treatments and with poorer survival [35,36]. Importantly, it can have implications regarding a patient’s ability to take medications appropriately (either chemotherapy or supportive medications) and may affect their ability to report side effects from treatment. Ensuring a patient has adequate cognitive abilities to understand their diagnosis and the proposed treatment is a vital element of determining capacity and obtaining informed consent. Therefore, providing care for people living with dementia presents ethical challenges. For example, comprehensive assessment must not only involve gauging the extent to which a patient is able to participate in their own treatment decision making, but also involving often one or more family members or caregivers. These persons may or may not have a legally appointed role for decision making and may have opinions which differ or conflict with the patient [37].

Cognitive decline has long been associated with cancer treatments, but also associated with a cancer diagnosis itself, making the older adult population already at risk of cognitive impairment even more vulnerable to this toxicity [38,39]. In a prospective study by Lange and colleagues, baseline cognitive function was assessed in 123 women over age 65 with a new diagnosis of early stage breast cancer. A total of 41% of the study population met criteria for baseline cognitive deficits after neuropsychological testing of episodic memory, working memory executive functioning and information processing, which is significantly greater than expected rates amongst the older adult population without a cancer diagnosis. To the contrary, Mandelblatt and colleagues also evaluated older adult women with early stage breast cancer in a prospective study that included an age-matched control group without cancer, and found that there was no baseline difference in cognitive function in the total study population [40]. But instead that only the subgroup of patients with more advanced stage (II and III) or with 2 or more medical comorbidities was strongly associated with cognitive impairment when compared with the control population. Taken together, these studies suggest that a cancer diagnosis itself (even prior to any systemic therapy) may be associated with decline in cognitive function particularly in a subset of patients, and oncologists should have a heightened concern and lower threshold for geriatric referral in these patients.

It is critical to identify pre-existing cognitive impairment in older patients with cancer prior to the initiation of any therapy, not just to ensure proper informed consent for treatment but also as a first step in managing and optimizing function and independence wherever possible while on treatment. While several tests exist, a streamlined screening tool, such as the Mini-Cog or Blessed-Orientation-Memory-Concentration test, can be used in an oncology clinic [41,42]. Patients who have a positive screening test should have further cognitive testing and/or assessment by a geriatrician or primary care provider for further evaluation. More in-depth tools such as the Mini-Mental Status Exam or Montreal Cognitive Assessment can also be used but are more time intensive. Cognition should be briefly reassessed with each subsequent line of systemic therapy for advanced disease, especially if considerable time has elapsed since the last assessment. If cognitive impairment is identified, patients (and their families) should be encouraged to pursue advanced care planning as early as possible, to maximize the ability of the patient to participate in all aspects of their own care planning.

Management strategies for cognitive impairment during treatment for advanced breast cancer has not been well-studied, and is inherently challenging as interventions must be highly individualized not only to the patient’s unique circumstance and deficits, but also based on availability of family and community-based health supports. Clinicians should consider closer follow-up, particularly at the beginning of each new line of therapy, and should have a low threshold for referral to community nursing and support groups. Clinicians should also encourage family and caregiver involvement wherever possible. Further research is needed to develop evidence-based and patient-centered interventions for management of cognitive impairment in patients with cancer, in an effort to optimize both cognitive and cancer-related outcomes in this vulnerable patient population.

### 4.3. Polypharmacy and Treatment Toxicities

Older adults are more likely to be on multiple medications, which can increase the risk of drug interactions, adverse effects, and treatment non-adherence. The prevalence of polypharmacy amongst older adults varies significantly in the literature, depending on the definition used, but remains a common concern. One recent study found a prevalence of 61% amongst a cohort of older adults starting treatment for advanced cancer in the United States [43]. The researchers found that with each additional medication, patients experienced an increasing risk of drug-drug interaction and drug-cancer treatment interaction, increasing the odds by 39% and 12% respectively. Older adults should have a dedicated pharmacy review for any potentially unnecessary medications or dangerous medication interactions at the time of their initial clinic consultation and with each subsequent line of therapy.

Older adults may experience distinct toxicities compared with their younger counterparts as well as potential differences in the timing of treatment toxicities. They may experience delayed or prolonged toxicities due to alterations in drug metabolism and clearance, as well as changes in body composition and organ function [44,45,46]. Close monitoring and dose adjustments may be necessary to mitigate these toxicities, aiming to strike a balance between maintaining treatment efficacy while optimizing patient safety and quality of life. In some cases, initiating treatment at a reduced dosage may be necessary to assess the patient’s tolerance. If treatment is well-tolerated, clinicians can then consider up-titration with future cycles. Dedicated tools, such as the Cancer and Aging Research Group (CARG) Chemo-Toxicity Calculator or Chemotherapy Risk Assessment Scale for High-Risk Patients (CRASH) tools, can help clinicians better estimate the risk of chemotherapy toxicity in patients aged 65 and over [36,47,48]. Additional tools to help predict toxicity in patients receiving targeted therapies, such as tyrosine kinase inhibitors, and immunotherapies, are needed.

The most recent guidelines from the International Society of Geriatric Oncology (SIOG) for the management of older adults with breast cancer emphasize the importance of supportive care through treatment, including special attention for digestive symptoms, malnutrition, depression, and pain control [13]. Proactive screening and management of treatment-related toxicity by skilled nursing or pharmacy staff (e.g., with telemedicine) may be helpful in supporting older adults on treatment [49]. In follow-up assessments, quality of life should be incorporated into evaluating treatment efficacy, as well as the potential impact of treatment on relevant geriatric syndromes (e.g., independence, cognition, mood, mobility, falls, incontinence). Lastly, supportive care with G-CSF primary prophylaxis should be considered in patients with higher risk of febrile neutropenia (FN), which might reduce related hospitalizations and dose reductions in older patients [50]. A lower FN risk threshold of 10%, instead of 20%, has been suggested for older adults [13].

### 4.4. Patient Values and Preferences

When considering treatment options and recommendations, it is essential to elicit and carefully consider each patient’s unique values, as well as their goals for their healthcare and interest in participating in clinical trials. Older adults may have varying perspectives and priorities shaped by their life experiences and overall health status. Living as long as possible may be important to some older adults, while quality of life and/or decreased treatment burden may be more important to others. Published results from a patient survey amongst older adults with hormone-sensitive breast cancer found that 44% of respondents were either “not comfortable” or “not interested” in participating in potential de-escalation clinical trials [51]. Another prospective comparison of younger and older patient preferences for systemic therapy in early stage breast cancer found distinct differences by age cohort. Older patients were less likely to accept chemotherapy but equally likely to accept endocrine therapy [52]. While older adults may value outcomes other than survival more often, age alone cannot predict this, and it is important to specifically ask patients what is important to them. Treatment plans should align with their voiced beliefs, and account for factors including functional status, preserving independence, and social and family supports. Ultimately, through a shared and active decision-making process, healthcare providers can ensure that treatment choices are congruent with the patient’s values and preferences, while respecting the fine balance between efficacy and toxicity.

It is important to acknowledge potential disparities in the access of older adults to oncologic care. The vulnerability that frailty imposes can be compounded with other important factors including racial and ethnic disparities in health care, socioeconomic status, disability, and geographic distance from treatment facilities [2]. Additionally, a patient’s physical environment may influence their treatment preference and opportunities. While geographically determined barriers to care are not uniquely experienced by older patients, challenges may be more profound when experienced together with declining independence or decreased mobility. For example, if a patient lives in a rural setting, they may encounter transportation barriers including greater drive time to and from cancer centres. If unable to drive themselves, they may worry about burdening family members, which may influence their treatment decisions. Similarly, patients in rural settings typically lack access to the same clinical trial opportunities as urban-residing patients, or instead at the cost of a greater personal time commitment traveling to trial-designated centres [53]. Patients with mobility restrictions likely experience compounded challenges in all these domains. Providers should be aware of these disparities and provide support through available resources and patient advocacy.

### 4.5. Palliative Care

Timely introduction to palliative care for older adults with metastatic cancer is recommended by the ASCO, European Society for Medical Oncology (ESMO), and SIOG guidelines [2,13,49]. However, as palliative care specialists are not always readily accessible, oncologists should be familiar with the fundamentals of palliative care, recognizing when expertise is required [54]. Broadly, treatment decisions should be informed by breast cancer risk factors as well as risk of dying from other causes [13]. These candid discussions may also help to reveal preferences in advanced care planning, including at end of life, which are issues often overlooked in routine oncology care but are highly valued by patients and caregivers [2]. To facilitate shared decision making, providers should openly communicate prognosis, treatment options, and expected outcomes with their patients. The ideal timing of advanced care planning is nuanced, but should take place when a metastatic diagnosis is identified, using a multidisciplinary approach (e.g., physicians, nurses, social work, and palliative care) [54]. A patient’s cultural and social values may be particularly important in these discussions, which should be respected and supported whenever possible.

## 5. Advanced Hormone-Sensitive, HER2-Negative Breast Cancer

Systemic therapy for advanced hormone-sensitive (HR+), HER2-negative breast cancer has evolved considerably over the last decade. Cyclin dependent kinase 4/6 (CDK 4/6) inhibitors combined with endocrine therapy (ET) are now standard of care in the first line setting based on results from three pivotal clinical trial programs evaluating the efficacy and safety of palbociclib, ribociclib and abemaciclib in this clinical context. All three approved CDK 4/6 inhibitors have demonstrated clinically meaningful and remarkably similar improvements in progression free survival (PFS) over endocrine therapy alone [55,56,57]. To date, only ribociclib has demonstrated overall survival (OS) benefit in the first line setting (MONALEESA 2, 3, and 7) [58,59,60]. Survival data for abemaciclib is not yet mature (MONARCH 3), and palbociclib failed to show a statistically significant improvement in OS over ET alone (PALOMA 2) [61]. While differences in outcomes as they relate to breast cancer (e.g., progression-free survival and overall survival) are important, when selecting the CDK4/6 inhibitor of choice, it is crucial to balance considerations of proven efficacy with the data available specifically in older adults and consider the unique side effects and toxicity profile of each medication (Table 1). Recently, results from the phase 3 SONIA trial challenged the assumption of using CDK4/6 inhibitor in the first line setting. No statistical difference in the second objective disease progression and overall survival were demonstrated if a CDK4/6 inhibitor was used in the first versus second line setting. While SONIA trial participants were mainly prescribed Palbociclib, this evidence must be taken into account when counselling patients about their treatment options so as to minimize unnecessary treatment and side effects. The results of this one trial must be balanced with the results of the trials of first line CDK4/6 inhibitors showing a consistent improvement in PFS and in some OS benefit.

All three trials included relatively similar numbers of older patients. Data on the proportion of patients over 70 or 75 years old is not consistently reported. In subgroup analyses of these older patient populations from all three trials, there was a consistent degree of PFS benefit with CDK 4/6 inhibitor compared with ET alone, regardless of age [62,63,64]. In PALOMA-2 patient reported function and quality of life were additionally reported and were maintained on treatment. PALOMA-2 also examined comorbidity in addition to age. A post-hoc subgroup analysis in patients with pre-existing comorbidity (41% of the total PALOMA-2 trial population) found preserved PFS benefit regardless of baseline pre-existing health conditions [65].

Safety data amongst the older adult patients in PALOMA-2 and MONALEESA-2 were generally consistent with the total trial population, whereas there were some more clinically relevant differences in tolerability seen in MONARCH-3. In MONARCH-3 there were higher dose reduction and discontinuation rates among older patients. Investigators also reported significantly higher rates of clinically relevant diarrhea (grade 2 and 3) amongst older patients receiving abemaciclib (<65, 39.5%; 65–74, 45.2%; ≥75, 55.4%). Rates of nausea, decreased appetite, and venous thromboembolism were slightly higher in older patient cohorts [64]. in PALOMA-2, reported adverse events were similar regardless of the presence, absence, or specific subtype of pre-existing comorbid condition. Similarly, real-world data consistently confirms efficacy and safety of palbociclib in older adults, and further suggests that the frequent dose-reductions required in this patient population outside of a clinical trial setting do not negatively impact outcomes [66,67,68].

A core limitation in interpreting these studies is that none included a detailed assessment of overall health and fitness at baseline. Notably, the majority of patients included in the studies were ECOG performance status 0–1, with only 2% of patients in the PALOMA-2 study being ECOG PS 2. It is likely that patients on these studies were highly selected with the majority of older adults being fit, cognitively intact and excluding patients with particular comorbidities (specifically a variety of cardiac comorbidities and arrhythmias). Real world data in older adult patients treated with palbociclib from the PALOMAGE program and P-REALITY X real-world analyses suggest consistent benefit with the PALOMA-2 results [69,70].

Despite these limitations, taken together, subgroup analyses from the above-described trials suggest that all three CDK 4/6 inhibitors can be safely used in an older adult patient population with HR+, HER2− ABC. Treatment decisions should remain tailored to individual patient concerns, comorbidities, and preferences. For instance, consider avoiding ribociclib in patients with pre-existing cardiac comorbidities or known QTc prolongation, while those with mobility concerns or gastrointestinal conditions may face challenges with abemaciclib-related diarrhea.

Despite improvements in breast cancer outcomes, there are circumstances in which single agent endocrine therapy may be appropriate and the SONIA trial has provided more data to support this approach. The primary outcome analysis from the SONIA trial suggests that PFS did not differ significantly in patients who received CDK 4/6 inhibitor in the second-line setting (given with fulvestrant) after prior endocrine monotherapy given first-line, compared with those who received upfront treatment with an aromatase inhibitor and CDK 4/6 inhibitor [71]. Factors including access to CDK 4/6 inhibitors in the second line setting and patient goals and preferences are important in applying data from this study.

The addition of a CDK 4/6 adds to pill burden and requires closer monitoring including blood work, EKGs, and more frequent clinical check-ups. Additionally, if a patient’s life expectancy is shorter than the expected progression free survival on endocrine therapy alone, they are unlikely to experience any benefit with the addition of a CDK 4/6 inhibitor. In such situations, after careful consideration and discussion with the patient, it is reasonable to treat this specific population with an aromatase inhibitor or fulvestrant alone.

Selecting subsequent lines of therapy for HR+, HER2-negative ABC in older adults necessitates careful re-evaluation of a patient’s evolving health and preferences, in particular their openness to treatments such as chemotherapy and treatments with higher rates of toxicity. Endocrine-directed treatments should be selected when possible, given their favourable toxicity profiles. Fit older patients should be considered for clinical trials, and for testing with a circulating tumour DNA next generation sequencing multigene panel to assess for actionable mutations. Based on these results, some patients may be eligible for targeted therapies such as alpelisib plus fulvestrant or capivasertib plus fulvestrant, if available. Although clinicians and patients must be aware of the potential for severe toxicities (e.g., diarrhea, hyperglycemia) with these targeted therapies.

Once endocrine therapy options are exhausted, fit older adult patients can be considered for later lines of sequential single-agent chemotherapy. While initial dose modifications are frequently required, single agent therapy with capecitabine is typically well-tolerated and effective, and represents a good cytotoxic treatment option in the older patient population with an attractive side effect profile (oral administration, no alopecia) [72]. Metronomic capecitabine is a well-tolerated alternative [73]. After exposure to chemotherapy, fit older patients may be considered for treatment with an antibody-drug conjugate such as trastuzumab deruxtecan if HER2-low (based on DESTINY-Breast04) or sacituzumab govitecan if HER2 0 by IHC (based on TROPiCS-02, although benefit was very modest) [74,75]. It is important to acknowledge that very few older patients, particularly those over the age of 70, were included in these studies. Of note, an age-specific pooled analysis of patients ≥ 65 years old who received trastuzumab deruxtecan found a favourable benefit-risk profile, with slightly increased toxicity [76]. Older adults who are not fit enough to receive chemotherapy can be considered for single agent fulvestrant or other oral endocrine therapies like tamoxifen. While benefit tends to be quite limited in this setting, these medications have a role in more frail patients who are interested in pursuing further treatment, have a strong preference for oral therapies, or in those who value quality of life over survival. Otherwise, frail patients in the endocrine-resistant setting should receive best supportive care.

## 6. Advanced HER2-Positive Breast Cancer

The incidence of HER2-positive (HER2+) breast cancer decreases with advancing age yet impacts a substantial proportion of patients diagnosed with breast cancer aged 70 or older, affecting approximately 11% of individuals in this age group [77]. Standard of care first-line treatment for HER2+ ABC was established in the pivotal CLEOPATRA trial publication. This trial showed significant improvement in both PFS (18.7 vs. 12.4 months, HR 0.68, 95% CI 0.58–0.80, *p* < 0.001) and OS (56.5 vs. 40.8 months, HR 0.68, 95% CI 0.56–0.84, *p* < 0.001) with addition of pertuzumab to docetaxel and trastuzumab [78].

Only 127/808 (16%) of patients in CLEOPATRA were ≥65 years old, and only 19/808 (2%) were ≥75 years old. While this makes it difficult to generalize the results to an older patient population, subgroup analyses from CLEOPATRA suggest consistent degree of benefit in PFS [79]. There were slight differences in toxicity profile. Older patients experienced more diarrhea, fatigue, asthenia, decreased appetite, vomiting, and dysgeusia compared with younger patients in both treatment arms. There was no significant association between age and development of left ventricular systolic dysfunction on treatment though patients with pre-existing cardiac comorbidities including low ejection fraction were excluded. Despite underrepresentation of older adults, these results suggest efficacy and safety of pertuzumab, docetaxel and trastuzumab in fit older adults and should be considered as the preferred first-line regimen for this population with HER2+ ABC. An excellent alternative for patients who may be too frail for docetaxel given every 3 weeks is weekly paclitaxel [80]. This alternative regimen has been validated as highly active and better tolerated and should be considered for any patient at increased risk of toxicity from chemotherapy. The main drawback being weekly travel for infusion appointments and increased chair time. Patients who have good response after 4–6 cycles of induction chemotherapy can continue on to trastuzumab plus pertuzumab alone to avoid further potential toxicities associated with chemotherapy.

For older patients who are too frail or have contraindications for taxanes, there are data from the HERNATA trial showing better tolerance of vinorelbine with trastuzumab compared with docetaxel and trastuzumab, with similar efficacy [81]. This is further supported by the Phase II VELVET study which showed efficacy and good tolerance of vinorelbine, pertuzumab and trastuzumab [82]. Whether this regimen is better tolerated compared with weekly paclitaxel, pertuzumab and trastuzumab is unknown. The EORTC 75111-10114 trial showed improved PFS in frail older patients with the addition of metronomic cyclophosphamide to trastuzumab and pertuzumab, compared with treatment with trastuzumab and pertuzumab alone [83].

There is also evidence for HER2-directed therapy alone without chemotherapy (with or without ET). The phase II PERNETTA trial found equivalent 2 year OS between patients receiving first line trastuzumab plus pertuzumab alone versus trastuzumab plus pertuzumab plus weekly paclitaxel or vinorelbine (both arms followed by second-line TDM-1) [84]. Applicability of these results are limited by short follow-up time. Results from the Phase II PERTAIN clinical trial also showed improvement in PFS with addition of pertuzumab to a backbone of trastuzumab plus ET in patients who did not receive any induction chemotherapy (mPFS 21.7 vs. 12.5 months, HR 0.55, 95% CI 0.34–0.88, *p* = 0.01) [85]. Patients were selected for induction chemotherapy based on physician discretion prior to randomization, and there was no formal comparison between those who received chemotherapy and those who did not. However, amongst all patients receiving treatment with pertuzumab, interestingly PFS was numerically higher in those who did not receive induction chemotherapy (21.7 vs. 16.9 months).

Endocrine therapy with or without trastuzumab (as anti-HER2 monotherapy) was also investigated in the TAnDEM trial for post-menopausal women with HR+, HER2+ ABC. Patients who received trastuzumab plus anastrazole versus anastrazole alone, experienced significantly improved PFS (4.8 vs. 2.4 months, HR 0.63, 95% CI 0.47–0.84, *p* = 0.0016) but no impact on OS [86]. There was no subgroup analysis by age. Endocrine therapy plus trastuzumab remains a possible option in frail older patients with HR+, HER2+ ABC, where chemotherapy is not appropriate and if funded provincially or through a patient’s private insurance.

Trastuzumab monotherapy remains an option for patients with hormone receptor-negative (HR-) ABC who are deemed too frail to undergo chemotherapy; however, its efficacy is significantly diminished when administered as a standalone treatment [87].

Following progression on first-line therapy, patients should be reassessed to determine fitness for subsequent lines. Fit older adults should be offered standard of care with trastuzumab deruxtecan [88]. Fit patients, especially those with brain metastases, should also be considered for treatment with tucatinib, trastuzumab and capectiabine, per the HER2CLIMB clinical trial [89]. Approximately 20% of the total trial population was over 65 years old, and subgroup analyses showed consistent benefit. Selection of second-line therapies and beyond for less fit older adult patients is challenging, as not only are treatments often taxing, but this patient population is very seldom included in clinical trials. Continued HER2 suppression is important whenever possible [90]. Patients not fit enough for treatment with an antibody-drug conjugate may be fit enough to receive continued trastuzumab with an alternate chemotherapy backbone. Table 2 summarizes the results of key trials relevant to the treatment of advanced HER2-positive breast cancer, and special considerations in older adults. 

## 7. Advanced Triple-Negative Breast Cancer

Triple-negative breast cancer (TNBC), which lacks expression of ER, PR, and HER2, is unique in its pattern of rapid growth and high recurrence risk with associated rapid decline, conferring a poorer prognosis than other subtypes of breast cancer with a median overall survival of less than 2 years [91,92,93]. TNBC disease biology is heterogenous, and so too can be the affected patients with regards to age, comorbidities, frailty, and social supports. Although more common amongst younger women, the incidence of TNBC among older adults remains significant, making up almost 10% of all breast cancers diagnosed in those over age 70 [77,92].

Older patients with TNBC are typically treated more conservatively compared with younger patients. Undertreatment of this population with systemic therapy has been identified as a reason why older patients, particularly those aged > 75, demonstrate poorer outcomes after adjusting for other high-risk factors [92]. A recent retrospective study of 524 patients with TNBC found that only 10% of older patients received palliative chemotherapy, compared with 93% of the younger group (*p* = 0.0004) [92]. Another retrospective study of nearly 500 patients aged 70+ with ABC (of whom 14% were TNBC) showed a higher risk of death with advanced age of 80–90 (HR 1.30, 95% CI 1.02–1.64; *p* = 0.028), ECOG performance status (PS) 3–4 (68% of the study population) (HR 2.34, 95% CI 1.73–3.15, *p* < 0.001), and not receiving systemic therapy (HR 4.48, 95% CI 2.88–6.98, *p* < 0.001) [94]. The need for more tolerable and acceptable treatments is apparent. These data underscore the difficulty in applying evidence from landmark clinical trials to the average older patient seen in clinic, as many patients in the real-world setting would never meet criteria for inclusion.

Due to the lack of therapeutic targets, chemotherapy remains the mainstay of treatment, aiming to prolong survival whilst controlling cancer-related symptoms. Historically, sequential single-agent chemotherapy has been the preferred treatment approach. Combination regimens are associated with more toxicity and no survival benefit [91]. There is no optimal or preferred sequence of agents; however, different treatment schedules, dose reductions or step-wise dose escalation may be required in older patients [13]. First-line chemotherapies for TNBC are usually chosen based on their toxicity profile, as well as a patient’s preferences, and fitness. While many of these agents have not been studied specifically in older patients, there are data supporting the use of nab-paclitaxel and eribulin in this setting [13,95,96].

More recently, with the results of KEYNOTE-355, pembrolizumab, a programmed death-1 (PD-1) inhibitor, has been approved for the first-line treatment of metastatic TNBC in combination with chemotherapy [97]. This improved OS in patients with a combined positive score (CPS) of ≥10 compared with chemotherapy alone (23.0 vs. 16.1 months, HR 0.73, CI 0.55–0.95, *p* = 0.0093) [98,99]. This benefit persisted among patients ≥ 65 with CPS ≥ 10 (28.3 vs. 12.6 months, HR 0.51, 95% CI 0.28–0.92). It is unclear how many patients were older than age 70 or 75, and notably all patients had an ECOG-PS of 0–1. The earlier IMpassion130 trial failed to show an improved OS in the intention-to-treat (ITT) population, as well as in patients aged ≥ 65, when comparing atezolizumab plus chemotherapy versus chemotherapy alone in the first-line treatment of metastatic TNBC [100].

While neither KEYNOTE-355 nor Impassion130 included a subgroup analysis for immune-related adverse events (IRAE) in the older population, available data suggest comparable IRAEs between older and younger patients [101,102]. Older adults, however, may not have the same functional reserve to recover from the potentially severe/life-threatening toxicity of immunotherapy. IRAE education, early detection, and timely intervention is important for all patients on immune checkpoint inhibitors; however, older adults may benefit further from careful identification of IRAE risk factors (e.g., high BMI, history of autoimmune conditions, elevated creatinine), and those that are modifiable should be optimized [91,103,104,105].

PARP-inhibitors have changed the treatment landscape for patients with germline BRCA mutations (gBRCAm) across multiple tumor sites, and these mutations are enriched in TNBC. In the OlympiAD trial of metastatic breast cancer in patients with gBRCAm, olaparib resulted in a longer mPFS (7.0 vs. 4.2 months, HR for progression or death 0.43, 95% CI 0.29–0.63, *p* < 0.001) but no OS benefit compared with chemotherapy in the TNBC subgroup [106]. Furthermore, only 5% of patients were >65 years old and the oldest patient was 76 [107]. There are limited data to support the efficacy and safety of olaparib in older adults, however this patient population should not be excluded from genetic testing based on chronological age alone given the implications for screening and familial testing [13,91].

Beyond first-line treatment for advanced TNBC, there is some evidence specific to older adults to inform treatment recommendations. The EMBRACE trial, which included patients up to age 80, demonstrated a modestly improved OS for metastatic patients treated with eribulin compared physician’s choice of chemotherapy [108]. A pooled analysis of three eribulin trials for metastatic disease showed comparable efficacy and safety outcomes in patients over and under 70 years of age; however, all included patients had to be fit enough for trial enrolment (i.e., ECOG-PS 0–2) [109]. There are little data about other factors, such as comorbidities, and how these affect treatment tolerance. The multicentric observational ERIBE study showed that older patients treated with eribulin had preserved quality of life on treatment [96].

Sacituzumab govitecan is an antibody-drug conjugate that has been approved for the treatment of patients with unresectable or metastatic TNBC who have received at least two prior therapies, with at least one in the metastatic setting [110]. The target of this agent, human trophoblast cell surface antigen 2, is expressed in 90% of TNBC [91]. In the phase III ASCENT trial, sacituzumab govitecan improved mPFS (5.6 vs. 1.7 months, HR 0.41, 95% CI 0.32–0.52, *p* < 0.001) and OS (12.1 vs. 6.7 months, HR 0.48, 95% CI 0.38–0.59, *p* < 0.001) compared with chemotherapy [111]. In a subgroup analysis of patients ≥65 years, mPFS remained significantly improved (7.1 vs. 2.4 months, HR 0.22, 95% CI 0.12–0.40) [20]. However, with a toxicity profile including more grade 3 diarrhea and myelosuppression compared with chemotherapy, added caution is advised for older, less-fit patients with consideration for dose reduction [91].

Metronomic chemotherapy has been shown to be acceptably effective and a well-tolerated alternative to conventional chemotherapy regimens in older adult populations with breast cancer [112,113,114]. The most commonly used chemotherapeutic agents appropriate for metronomic dosing are capecitabine, cyclophosphamide, methotrexate and vinorelbine—and combinations of these medications have also been studied. Generally, metronomic administration is considered a safe, effective, and easy to titrate alternative for patients unsuitable for conventional bolus dosing.

While several advances in TNBC have been made recently, there are limited data in older adults, particularly those in their 70’s and 80’s, and the majority of experience thus far has been in patients with good performance status and limited comorbidities. Based on experience and the breadth of prior data, in general, fit older adults should be treated with standard of care therapies, frail patients should be considered for best supportive care, and those in between can be considered for modified treatment (e.g., upfront dose reduction or metronomic administration) and likely require closer monitoring. Table 3 summarizes the results of key trials relevant to the treatment of advanced triple-negative breast cancer, and special considerations in older adults.

## 8. Discussion

Older adults with advanced breast cancer face many unique challenges and may have highly variable treatment preferences and goals. It is imperative that clinicians make treatment recommendations based on physical and cognitive fitness, and not chronological age. Table 4 summarizes key considerations and treatment approaches for older adults with ABC, by biomarker profile and degree of frailty.

As discussed throughout this paper, there is often limited data regarding the efficacy, safety and tolerability of both standard of care and novel emerging therapeutic options in the older adult patient population. Table 5 highlights active studies, mostly interventional, assessing clinical outcomes in the treatment of older adults with advanced breast cancer using standard of care treatment options. In this patient population, safety is the most commonly used primary endpoint in current research. As discussed, the enrolment of adults aged 70 and older has been limited in the landmark studies informing treatment guidelines. Therefore, the results of these studies should help to bridge a knowledge gap in clinical practice.

Revisiting the clinical case of Ms. L, the independent 91-year-old female recently diagnosed with ABC. Biomarker profile from the breast biopsy returns showing ER 8/8, PR 8/8, and HER2 1+ by IHC. Staging investigations confirm bone-only metastatic disease. At initial consultation, brief GA assessment using the G8 Geriatric screening tool yields a score of 15, indicating comprehensive geriatric evaluation not necessary at that time. After counselling about available therapies and relevant data, including results from the recent SONIA clinical trial, she was considered for first-line ribociclib and letrozole, but as she was found to have QTc prolongation the prescriptions was changed to palbociclib plus letrozole. She was started at full dose palbociclib at 125 mg daily, with subsequent dose reduction to 100 mg daily due to neutropenia with good tolerance.

After 2 cycles of palbociclib (to ensure she has tolerated the treatment well), she started treatment with an IV bisphosphonate for prevention of skeletal-related events.

Ms. L experiences stable disease for 3 years on palbociclib and letrozole but during this time, her functional abilities and independence have declined. She now walks with a 4-wheeled walker, and recently moved into an assisted living facility. She voices no concerns about bone pain, but just generalized weakness. She has a close relationship with her family physician and has recently been seen in a multidisciplinary specialty geriatrics clinic. She now requires help with all iADLs, in part due to declining memory and cognitive function with a recent mini-Cog score of 1/5 in clinic (1 word recall only). A re-evaluating CT scan now shows several small new liver lesions. She is asymptomatic. You discuss the results of the scan and ask about Ms. L’s goals and priorities for her life and healthcare. She shares she would like to focus on her quality of life and to minimize medical appointments. She is particularly concerned about the impact of treatment-induced side effects on her function and quality of life. There is no public funding for fulvestrant monotherapy in her jurisdiction, and she does not have extended health benefits. In light of this, you collectively decide to switch to second-line tamoxifen. You frankly discuss that there is little chance of prolonged response to tamoxifen alone in second-line. Ms. L is realistic in her outlook, and appreciates that beyond tamoxifen she will be offered best supportive care (based on her voiced preferences plus functional status).

It is critical her healthcare team broach advanced care planning including end of life care, and if appropriate to share these preferences with her family or identified substitute decision maker. You advise her to ensure her will and her affairs are in order. This discussion would have been appropriate many years prior, but becomes even more pressing given the recent changes in her health.

It is important to consider how Ms. L’s clinical course may differ if she lived in a rural environment. Each in-person appointment or diagnostic investigation would involve a longer travel time, and perhaps a companion or driver to each appointment. The two particular lines of therapy detailed in this case can be relatively easily managed and monitored in a rural-residing patient, given they are orally administered. However, if Ms. L had a HER2+ or triple-negative biomarker profile, or had HR+ ABC but was fit and wanted to proceed to sequential single-agent chemotherapy, she would require more in person visits which would increase the time commitment for travel. This can meaningfully impact a patient’s quality of life and may influence their decision-making regarding treatment selection. This reinforces the critical role that telemedicine and distributed community-based infusion units play in breaking down some of the barriers experienced by rural-residing patients with cancer.

## 9. Conclusions

Older adults living with advanced breast cancer represent a highly diverse patient population. Optimal treatment selection must reflect both physical and cognitive function and fitness, and incorporate patient values and preferences. Fit patients should be offered standard of care regardless of chronological age, while vulnerable and frail patients should have their health optimized if possible and be considered for modified treatment options (either alternative treatments or dose modifications) and/or more frequent monitoring. Patients of all ages should have specific opportunity to share their values and preferences for their healthcare. Furthermore, patient fitness and values assessments must be considered dynamic, and frequently re-evaluated (at a minimum each time therapy is changed).

## Figures and Tables

**Table 1:** Summary of key trials and considerations for use of CDK 4/6 inhibitors in older adults.

CDK 4/6 Inhibitor	mPFS	mOS	Key Toxicities	Differential Effects in Older Adults
Palbociclib (P): PALOMA-2 compared P + letrozole (L) vs. L	24.8 (P + L) vs. 14.5 (L) mo, HR 0.58, 95% CI 0.46–0.72	No statistically significant difference	Myelosuppression Infection Fatigue Nausea Rare risk of interstitial lung disease	39% of total trial population were ≥ 65 years old. Pooled analysis in patients ≥ 65 showed similar significant improvements in PFS. Preserved PFS benefit in those with comorbid illness (41% of PALOMA-2 population). Patients ≥ 75 had more myelosuppressive toxicity.
Ribociclib (R): MONALEESA-2 compared R + L vs. L	25.3 (R + L) vs. 16 (L) mo, HR 0.57, 95% CI 0.46–0.70	63.9 (R + L) vs. 51.4 (L) mo, HR 0.76, 95% CI 0.63–0.93	Myelosuppression Infection Fatigue Nausea Rare risks of hepatic toxicity and QTc prolongation	44% of total trial population were aged ≥65. Subgroup analysis in these patients showed consistent PFS benefit and safety profile.
Abemaciclib (A): MONARCH-3 compared A + L vs. L	28.2 (A + L) vs. 14.8 (L) mo, HR 0.54, 95% CI 0.42–0.7	Not mature	Diarrhea Nausea Fatigue Myelosuppression Infection Transaminitis Rare risk interstitial lung disease and venous thromboembolism	45% of total trial population were aged ≥65. Consistent PFS benefit regardless of age. Significantly higher grade 2 or 3 diarrhea in older patients (<65, 39.5%; 65–74, 45.2%; ≥75, 55.4%). Higher risk of nausea, decreased appetite and VTE in older adults. Neutropenia rates did not differ by age.

Abbreviations: mPFS: median progression free survival; mOS: median overall survival; mo: months; HR: hazard ratio; CI: confidence interval; VTE: venous thromboembolism.

**Table 2:** Summary of key trials in advanced HER2-positive breast cancer and considerations for treatment in older adults.

Clinical Trial and Regimen	mPFS	mOS	Key Toxicities	Differential Effects in Older Adults
CLEOPATRA: Docetaxel (D) + Trastuzumab (T) + Pertuzumab (P) vs. D + T in 1 L setting	18.7 (D + T + P) vs. 12.4 (D + T) mo, HR 0.68, 95% CI 0.58–0.80	56.5 (D + T + P) vs. 40.8 (D + T) mo, HR 0.68, 95% CI 0.56–0.84	Older patients experienced more diarrhea, fatigue, asthenia, decreased appetite, vomiting and dysgeusia compared with younger patients. Pre-existing cardiac comorbidities were excluded.	16% of total trial population were ≥65 years old, 2% were ≥75. Subgroup analysis in patients ≥ 65 showed similar significant improvements in PFS. Consider weekly paclitaxel instead of docetaxel to minimize toxicity from chemotherapy.
VELVET (Phase II): Vinorelbine, trastuzumab and pertuzumab in 1 L setting	14.3 mo, 95% CI 11.2–17.5	Not reached. 2-year OS was 78.3%	Diarrhea Neutropenia Leukopenia Nausea Asthenia Decreased LVEF	Median age 56. Oldest patient on trial 82. Well-tolerated. Useful in patients with contraindications for taxanes. No direct comparison with weekly paclitaxel, trastuzumab and pertuzumab.
DESTINY-Breast03: trastuzumab deruxtecan (T-DXd) vs. trastuzumab emtansine (T-DM1) in 2 L setting	28.4 (T-DXd) vs. 6.8 (T-DM1) mo, HR 0.33, 95% CI 0.26–0.43	Not reached	Nausea Fatigue Vomiting Alopecia Neutropenia Thrombocytopenia Leukopenia ILD/Pneumonitis	18.8% of total trial population were aged ≥65. Similar PFS benefit regardless of age. Higher rate of serious adverse events in patients ≥ 65 vs. < 65 (32.2 vs. 24.3%). Any grade ILD was 11.8% in <65, 17.5% in patients ≥ 65 [76].
HER2CLIMB: tucatinib + trastuzumab + capecitabine (TTC) vs. trastuzumab + capecitabine (TC)	7.8 (TTC) vs. 5.6 (TC) mo, HR 0.54, 95% CI 0.42–0.71	24.7 (TTC) vs. 19.2 (TC) mo, HR 0.73, 95% CI 0.59–0.90	Diarrhea Hand-foot syndrome Nausea Fatigue Vomiting	19% of trial population over 65. Subgroup analysis showed consistent benefit. Safety data not differentially reported by age.

**Table 3:** Summary of key trials in advanced triple-negative breast cancer and considerations for treatment in older adults.

Clinical Trial and Regimen	mPFS	mOS	Key Toxicities	Differential Effects in Older Adults
KEYNOTE-355: Pembrolizumab (P) + treatment of physicians choice chemotherapy (TPC) vs. TPC in 1 L setting for patients with combined positive score (CPS) ≥ 10	9.7 (P + TPC) vs. 5.6 (TPC) mo, HR 0.66, 95% CI 0.50–0.88	23.0 (P + TPC) vs. 16.1 (TPC) mo, HR 0.73, 95% CI 0.55–0.95	Autoimmune thyroid disorders Pneumonitis Colitis Cytopenias Nausea Fatigue Rash	Trend for PFS improvement consistent among patients ≥ 65. Unclear how many patients were older than 70. Other data suggest rates of immune-related adverse events are similar among older patients.
ASCENT: sacituzumab govitecan (SG) vs. chemotherapy treatment of physician’s choice (TPC) in 2 L+ setting	5.6 (SG) vs. 1.7 (TPC) mo, HR 0.41, 95% CI 0.32–0.52	12.1 (SG) vs. 6.7 (TPC) mo, HR 0.48, 95% CI 0.38–0.59	Neutropenia Leukopenia Diarrhea Febrile neutropenia	In subgroup ≥ 65, PFS remained significantly improved. Older adults had more grade 3 diarrhea and myelosuppression. Caution in older less fit patients.
EMBRACE: eribulin (E) vs. treatment of physician’s choice (TPC) chemotherapy (TPC) in 3 L+ setting	3.7 (E) vs. 2.2 (TPC) mo, HR 0.87, 95% CI 0.71–1.05	13.1 (E) vs. 10.6 (TPC) mo, HR 0.81, 95% CI 0.66–0.99	Asthenia Fatigue Neutropenia Leukopenia Peripheral neuropathy	Included patients up to age 80. Pooled analysis showed equivalent efficacy in patients both over and under 70 years old. All patients ECOG 0–2.

**Table 4:** Summary of the key considerations when selecting treatment for older adult patients with advanced breast cancer (ABC), subdivided by biomarker profile and degree of frailty.

Consider All Older Adult Patients for Brief Geriatric Assessment in the Oncology Clinic			
Understand and grade the severity of frailty. Does the patient need a comprehensive geriatric assessment?	**Fit** 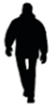	**Vulnerable** 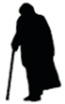	**Frail** 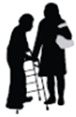
Core treatment considerations	Fit older adult patients should be offered standard of care. Encourage clinical trial participation. Review other medications to check for interactions. Discuss goals of care.	Address vulnerabilities identified to help improve tolerability of proposed therapy. Consider tailoring dose or frequency of medications. Institute supportive measures if needed. Merits close monitoring while on active therapy and dose modifications as needed. Review other medications to check for interactions. Discuss goals of care.	Consider alternative treatments including best supportive care. Start with initial dose reductions. Close monitoring while on active therapy with dose modifications as needed. Review other medications to check for interactions. In depth discussion about quality of life on treatment and goals of care.
Hormone-sensitive, HER2-negative ABC 1 L systemic therapy	Standard of care therapy with CDK 4/6 inhibitor plus AI at standard dosing level at initiation. Encourage clinical trial participation. Dose reduce as necessary. Can consider upfront single agent AI.	Consider initial empiric dose reduction in CDK 4/6 inhibitor, and up titrate if tolerating well. Careful monitoring with early cycles. Monotherapy with AI is also reasonable if this aligns with the patient’s values and preferences.	Consider AI monotherapy if aligns with the patient’s values and preferences. Best supportive care can be appropriate.
HER2-positive ABC 1 L systemic therapy	Offer standard of care with trastuzumab, pertuzumab, and taxane chemotherapy. Encourage clinical trial participation.	Consider weekly paclitaxel with trastuzumab and pertuzumab. Can empirically dose reduce paclitaxel. Low threshold to drop taxane if side effects that may precipitate functional decline. Can alternatively consider vinorelbine or metronomic cyclophosphamide plus trastuzumab and pertuzumab.	Consider best supportive care. Alternative options include: trastuzumab plus endocrine therapy in triple positive biomarkers if accessible. Or endocrine therapy alone if HER2 directed therapy is not available. Trastuzumab plus pertuzumab or trastuzumab monotherapy are both potential options, although efficacy is limited.
Triple-negative ABC 1 L systemic therapy	Assess PDL-1 status and offer standard of care pembrolizumab plus chemotherapy is CPS ≥ 10, versus chemotherapy alone if CPS < 10. Encourage clinical trial participation.	Assess PDL-1 status and carefully consider if fit enough for chemoimmunotherapy versus chemotherapy alone. Consider empiric dose reductions. Frequent monitoring and nursing visits	Consider best supportive care.

**Table 5:** Summary table of current studies assessing clinical outcomes specifically in older adults with advanced breast cancer from https://clinicaltrials.gov (accessed on 1 December 2023). HR = hormone receptor; HER2 = human epidermal growth factor receptor 2; ER = estrogen receptor; M = male; F= female; ABC = advanced breast cancer; LHRHa= luteinising hormone-releasing hormone agonist.

NCT Number	Design	Title	Status	Population	Intervention	Primary Endpoint	Study Hypothesis/Primary Objective
NCT03956654	Observational, prospective cohort	A Phase IV Study to Collect Data on the Efficacy and Safety of RIBociclib in Older Women With Breast cancer (RibOB)	Active, not recruiting	F aged 70+ with HR+/HER2− ABC	Ribociclib and letrozole	Progression free survival	This study evaluates the clinical efficacy, overall safety and tolerability of ribociclib in combination with letrozole in older women (≥70 years) with HR+/HER2− ABC and no prior hormonal treatment for advanced disease
NCT03944434	Interventional, phase II	FACILE: FeAsibility of First-line riboCIclib in oLdEr Patients With Advanced Breast Cancer (FACILE)	Active, not recruiting	M/F aged 70+ with HR+/HER2− ABC	Ribociclib and AI or LHRHa in males	Treatment feasibility	This study assesses the feasibility of first line ribociclib in combination with a non-steroidal AI in women or men aged 70 years-old or older, with HR+/HER2− ABC
NCT03633331	Interventional, phase II	Palbociclib and Letrozole or Fulvestrant in Treating Patients With Estrogen Receptor Positive, HER2 Negative Metastatic Breast Cancer	Active, not recruiting	M/F aged 70+ with HR+/HER2− ABC	Palbociclib and letrozole or fulvestrant	Incidence of adverse events	This study estimates the safety and tolerability (adverse event rate) of the combination of palbociclib and letrozole or fulvestrant in adults age 70+ with ER+/HER2− metastatic breast cancer
NCT03477396	Interventional, phase II	Ribociclib and Aromatase Inhibitor in Treating Older Participants With Hormone Receptor Positive Metastatic Breast Cancer	Active, not recruiting	M/F aged 70+ with HR+/HER2− ABC	Ribociclib and AI	Number of participants with grade 2+ toxicities attributed to ribociclib	This study estimates the safety and tolerability of the combination of ribociclib and an AI in adults age 70+ with HR+/HER2− ABC
NCT01273610	Interventional, phase II	Tolerability of the Combination of Lapatinib and Trastuzumab in Adults Age 60 or Older With HER2-Positive Locally Advanced or Metastatic Breast Cancer	Active, not recruiting	M/F aged 60+ with HER2+ locally advanced or metastatic breast cancer	Lapatinib and trastuzumab	Percent of participants with grade 3+ non-hematological toxicities and symptomatic congestive heart failure	To estimate the safety and tolerability of the combination of trastuzumab and lapatinib in adults age 60+ with locally advanced or metastatic breast cancer
NCT06044623	Interventional, phase III	Implementing Geriatric Assessment for Dose Optimization of Cyclin-dependent Kinase (CDK) 4/6-inhibitors in Older Breast Cancer Patients (IMPORTANT)	Not yet recruiting	M/F aged 70+ with HR+/HER2− ABC	Geriatric assessment to inform CDK4/6i doses	Time to treatment failure	The study hypothesis is that adjusting the CDK4/6i dose according to vulnerability will allow patients to tolerate treatment better without jeopardizing the treatment efficacy

## Data Availability

No new data were created or analysed in this study. Data sharing is not applicable to this article.
